# Defining an Accelerated Rehabilitation Protocol Following Anterior Cruciate Ligament Reconstruction: A Scoping Review

**DOI:** 10.3390/medicina62071348

**Published:** 2026-07-12

**Authors:** Maximilian Heinz, Jonathan Lettner, Aleksandra Królikowska, Maciej Daszkiewicz, Sebastian Damm, Nikolai Ramadanov, Roland Becker, Robert Prill

**Affiliations:** 1 Center of Orthopedics and Traumatology, University Hospital Brandenburg/Havel, Brandenburg Medical School, Hochstraße 29, 14770 Brandenburg an der Havel, Germany; 2 Faculty of Health Science, Brandenburg Medical School, 14770 Brandenburg an der Havel, Germany; 3 Physiotherapy Research Laboratory, University Centre of Physiotherapy and Rehabilitation, Faculty of Physiotherapy, Wroclaw Medical University, 50-372 Wroclaw, Poland

**Keywords:** anterior cruciate ligament reconstruction, accelerated rehabilitation, criterion-based rehabilitation, weight-bearing, return to sport

## Abstract

*Background and Objectives*: Accelerated rehabilitation after anterior cruciate ligament reconstruction (ACLR) is widely implemented, yet its definition and distinguishing characteristics remain inconsistently described in the literature. This scoping review examined how accelerated rehabilitation after ACLR is defined, described common protocol features, and identified elements distinguishing it from conventional rehabilitation. *Materials and Methods*: A scoping review was conducted using systematic searches of Medline (PubMed), Embase, and Web of Science from 1 April 1967 to 26 October 2025. Studies including patients aged 16 years or older who underwent primary ACLR that reported any form of accelerated rehabilitation or early progression relative to conventional protocols were eligible for inclusion. *Results*: Of 6002 screened records, 64 studies met the inclusion criteria. Accelerated rehabilitation was consistently characterized by early restoration of knee range of motion, early full weight-bearing, rapid gait normalization, early initiation of closed and open kinetic chain exercises, and avoidance of prolonged immobilization. However, definitions varied substantially across studies. Substantial heterogeneity was observed in progression timelines, bracing and crutch use, and return-to-sport criteria. *Conclusions*: Accelerated rehabilitation after ACLR appears to represent a brace-free, criterion-based, function-oriented approach emphasizing early restoration of knee extension, progressive loading, and individualized progression rather than simply shortened timelines. Establishing consensus definitions and standardized reporting is necessary to improve comparability across studies and facilitate translation into clinical practice.

## 1. Introduction

Anterior cruciate ligament (ACL) tears are common injuries, and reconstruction remains a commonly performed treatment option. It is estimated that more than 100,000 ACL reconstructions (ACLR) are performed annually in the United States [1,2,3]. Given this incidence, optimizing postoperative rehabilitation remains critical. In this traditional paradigm, rehabilitation protocols often involved 6–8 weeks of immobilization followed by slow progression of weight-bearing (WB). Such prolonged immobilization was later recognized to cause complications: patients often developed arthrofibrosis, loss of full extension, anterior knee pain, extensor mechanism dysfunction, and marked quadriceps atrophy [4]. Consequently, these aspects may be associated with poor return to sport (RTS) outcomes.

In 1990, Shelbourne and Nitz introduced an accelerated rehabilitation protocol in which patients began moving the knee fully into extension and immediately began full WB after surgery [5]. This paradigm shift demonstrated that early mobilization does not appear to compromise graft stability and could be associated with improved functional outcomes [6].

Preventing complications such as arthrofibrosis or loss of full extension is also critical. Studies suggest that failure to regain full extension early predisposes patients to a stiff knee; conversely, an aggressive focus on achieving knee extension from the outset substantially reduces the incidence of flexion contracture [4,7].

While some progressive rehabilitation principles have been implemented in clinical practice, there is inconsistency in terminology and rationales [8].

Currently, criterion-based progression utilizing objective strength measurements and functional assessments has widely accepted for determining readiness to RTS [9]. The purpose of this scoping review is to identify how “accelerated rehabilitation” after ACLR is defined in the literature, to determine common characteristics among reported protocols, and to propose a clearer, more consistent use of the term for future research and clinical practice.

## 2. Materials and Methods

### 2.1. Protocol and Registration

For the reporting of this scoping review, we followed the Preferred Reporting Items for Systematic Reviews and Meta-Analyses extension for Scoping Reviews (PRISMA-ScR) (Supplementary Material) and the Joanna Briggs Institute (JBI) Author Guidelines for Conducting Scoping Reviews [10,11,12]. This review was preregistered on Open Science Framework (osf.io/7ymt9).

### 2.2. Inclusion Criteria

Studies were selected for inclusion based on the following eligibility criteria: (1) male or female participants aged ≥16 years who underwent primary ACLR; and (2) studies reporting a rehabilitation protocol demonstrating any form of accelerated progression relative to conventional rehabilitation after ACLR, regardless of terminology used, provided it was evident that the protocol promoted a faster recovery or earlier initiation of activities. There were no restrictions regarding the year of publication. Articles were eligible for inclusion if an abstract was available and the language was accessible to the review team (German, Polish, or English). Studies were excluded if they constituted non-original publications (editorials, letters, reviews, duplicate reports, case reports, surgical technique description, and commentaries). When full texts were not accessible, the corresponding authors were contacted. Studies were excluded if the full text could not be obtained.

### 2.3. Search Strategy

A comprehensive literature search was performed in Medline (PubMed), Embase, and Web of Science for studies published from 1 April 1967 to 26 October 2025. The following search terms were used: (“anterior cruciate ligament reconstruction”[Title/Abstract] OR “ACL reconstruction”[Title/Abstract] OR “anterior cruciate ligament”[Title/Abstract]) AND (“rehabilitation”[Title/Abstract]). The search query was adapted slightly for each database to maximize retrieval coverage.

### 2.4. Screening and Selection of Sources of Evidence

All identified articles were imported into Zotero (Corporation for Digital Scholarship, Vienna, VA, USA, 2024) for reference management, and duplicates were removed. Two reviewers (M.H. and S.D.) independently screened the literature using the Rayyan (Qatar Computing Research Institute, Doha, Qatar, 2024) web application [13]. The screening process was divided into an initial review of title and abstract, followed by full-text evaluation of potentially relevant articles. At each stage, studies were assessed against the predefined eligibility criteria. In cases of disagreement, a more experienced third reviewer (J.L.) was consulted to reach a consensus regarding inclusion or exclusion.

### 2.5. Data Extraction and Descriptive Synthesis

Data extraction was performed independently by two reviewers (M.H. and S.D), with discrepancies resolved through discussion and, if necessary, consultation of a third reviewer (J.L.). The following data were extracted: first author, year of publication, country of origin, journal, study design, study population, sex, age, patient characteristics, and a detailed description of the rehabilitation protocol. The primary outcome of interest was the content and structure of accelerated rehabilitation protocols following ACLR. Rehabilitation protocols were systematically analyzed by extracting predefined rehabilitation domains, including WB progression, range-of-motion (ROM) progression, strengthening exercises, neuromuscular training, criteria for return to running, swimming, and cycling, and RTS criteria. Within each domain, rehabilitation characteristics, their timing, progression, and prescribed criteria were identified, grouped, and synthesized across studies. The synthesized data were subsequently presented in tabular and narrative form to compare rehabilitation strategies and identify common patterns, variations, and evidence gaps.

## 3. Results

### 3.1. Search Results

**Figure 1:**
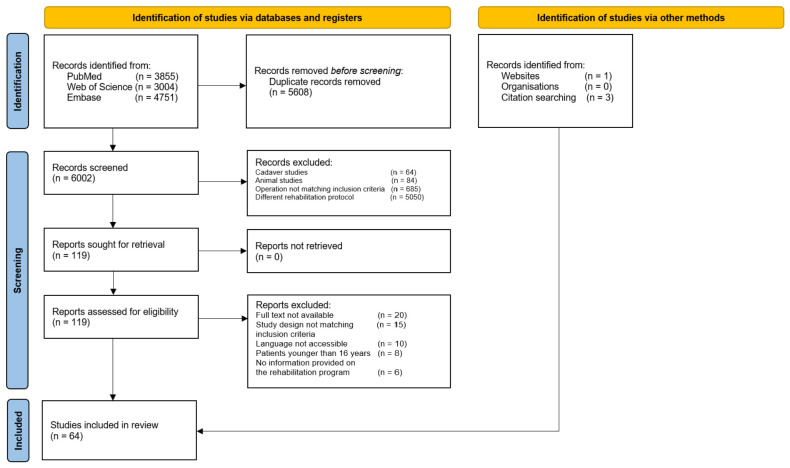
PRISMA Flow Diagram of Study Selection.

A total of 11,610 studies were identified based on the predefined search criteria. After the removal of 5608 duplicates, 6002 unique records remained for screening. During the title and abstract screening, 5856 records were excluded as they did not meet the inclusion criteria. Of the 119 articles assessed for eligibility, 44 were excluded for the following reasons: full text was unavailable for 20 studies despite contacting the authors; 15 studies designs did not match the inclusion criteria; 10 studies were not available in the prespecified eligible languages; 8 studies included patients younger than 16 years; and 6 studies did not provide sufficient information about their rehabilitation protocol. Four studies were identified through additional methods: one via the Umeå University website and three through manual citation searching (screening reference lists and citing articles of included studies). Ultimately, 64 studies with 4783 patients and a mean age of 27.5 years were included in this scoping review. A detailed overview of the study selection process is presented in the PRISMA flow diagram (Figure 1). The baseline characteristics of the included population are presented in Table 1.

### 3.2. Preoperative Phase

The preoperative phase primarily aims to restore full ROM and resolve swelling in the injured knee [22,77,78,79]. However, only a limited number of studies have examined this phase within accelerated rehabilitation protocols following ACLR. Multiple authors emphasize that achieving full ROM, particularly terminal extension, and physiological hyperextension matching the contralateral knee are essential, along with minimizing swelling and normalizing gait and lower-limb neuromuscular control [21,22,77,78]. Strengthening exercises targeting a limb symmetry index (LSI) of at least 75% prior to surgery are recommended to optimize muscle function. Overall, the literature suggests that pain, inflammation, swelling, ROM, and neuromuscular control, including gait mechanics, should be optimized before surgery. Moreover, providing clear preoperative instructions on postsurgical exercises and the use of crutches may enhance patient self-efficacy and support early postoperative recovery [41].

### 3.3. Early Phase Management: Knee Brace Use, ROM Restoration, and Weight-Bearing

An overview of the early postoperative rehabilitation components extracted from the included studies is presented in Table 2. The following sections describe the individual rehabilitation domains in detail.

#### 3.3.1. Knee Brace Protocols

Brace use varies markedly among studies, ranging from no bracing to 12 postoperative weeks (POW) of immobilization. Many accelerated protocols advocate eliminating the brace entirely [27,35,38,47,48,49,50,61,73,74], allowing unrestricted ROM from postoperative day (POD) 0-1. Short-term bracing from POW 1-4, often with restricted ROM of 0° or 0–60°, is also common [5,14,15,16,17,28,29,34,36,41,43,44,45,52,53,54,56,58,63,65,66,67,69,75,76], with transition to functional bracing typically occurring by POW 2-6 [5,14,16,17,18,28,29,31,32,36,45,46,53,57,63]. Prolonged bracing more than six weeks appears primarily in more restrictive protocols [52,55].

#### 3.3.2. ROM Restoration: Extension and Flexion Goals

Most protocols emphasize early restoration of full knee extension combined with a gradual progression of flexion to protect the graft while minimizing stiffness. Unrestricted extension is often allowed from POD 0-2 [5,14,15,16,17,18,20,21,22,26,28,29,33,36,37,38,40,41,45,47,48,49,50,53,54,58,60,61,63,64,71]. Flexion is usually progressed more cautiously to protect the graft and minimize anterior knee irritation, starting with a restricted range of about 90° during POW 1-2 and then increasing stepwise to 120–135° and eventually full flexion by around POW 4-6 [5,14,15,16,17,18,20,21,22,28,29,31,34,36,37,38,40,41,43,45,46,47,48,49,50,52,53,54,57,58,59,60,61,62,63,64,65,67,69,70,71,72,75].

#### 3.3.3. Weight-Bearing Progression

Full WB after surgery is recommended in most protocols by POD 0-2 [5,14,15,16,17,18,19,20,21,22,23,25,26,27,28,29,30,31,36,37,38,39,40,41,42,45,47,48,49,50,53,54,55,59,60,61,62,63,68,69,71,72,73,74]. Other protocols introduce short delays, such as permitting full WB between POD 1-5 or within POW 1-2 [24,32,33,34,46,51,57,58,64,65,66,67,70]. Prolonged restrictions lasting up to POW 3-9 are uncommon and typically coincide with conservative bracing strategies [43,44,52,56,75,76]. The use of crutches generally mirrors WB progression, with most protocols discontinuing crutches between POD 0-7, while more cautious protocols extend their use to POW 1-4.

### 3.4. Exercise Modalities: Continuous Passive Motion, Kinetic Chain Exercises, and Additional Therapies

The exercise modalities reported across the included rehabilitation protocols are summarized in Table 3. The following sections describe the reported use of continuous passive motion (CPM), closed kinetic chain (CKC) exercises, open kinetic chain (OKC) exercises, and adjunctive therapies [58,64,67,69].

#### 3.4.1. Continuous Passive Motion Protocols

CPM is not routinely incorporated into rehabilitation protocols and, when applied, was generally restricted to POW 1-2. The ROM was commonly limited, most frequently between approximately 0–30° or 0–90°. CPM was typically initiated on POD 0-1 and discontinued once active ROM exercises and functional training were advanced, indicating no essential role within contemporary ACLR rehabilitation strategies [5,14,16,17,21,22,23,28,29,36,40,43,45,47,48,49,50,53,55,58,59,61,62,63,67,68,69,71].

#### 3.4.2. Implementation of Open Kinetic Chain Exercises

OKC exercises were predominantly introduced early in the rehabilitation process and mainly consisted of supine or seated, quadriceps-dominant activities such as straight leg raises, short-arc knee extensions, and isometric knee extensions. These exercises were frequently initiated between POD 0-2 [5,14,15,16,17,18,19,21,22,23,28,29,31,36,38,40,43,45,53,55,58,59,60,62,63,69,71,72,74,75]. More dynamic OKC knee extension and flexion exercises showed a longer delay before initiation at POW 2 up to 8, reflecting a cautious approach aimed at balancing early quadriceps strengthening with concerns regarding anterior tibial translation and graft loading [18,32,37,41,46,57].

#### 3.4.3. Implementation of Closed Kinetic Chain Training

CKC exercises represented a core component of nearly all rehabilitation protocols and were commonly initiated within the first POW. Early CKC activities typically included low-load exercises such as wall slides, quarter squats, calf raises, and step-ups. Progression to higher-demand CKC exercises, including leg press, half or full squats, lunges, lateral shuffle drills, and stairmaster generally occurred between POW 2 and 6, in parallel with improvements in ROM and WB tolerance [5,14,15,16,17,18,19,21,22,23,28,29,31,36,38,40,43,45,53,55,58,59,60,62,63,69,71,72,74,75].

### 3.5. Sport-Specific Training: Jogging, Cycling, Swimming and Return to Sport

Sport-specific training after ACLR was introduced with distinct but heterogeneous timelines for jogging, cycling, swimming, and RTS across the included accelerated protocols, as presented in Table 4. Overall, time-based progression was frequently combined with objective, performance-oriented criteria such as strength and hop-test symmetry to guide safe advancement.

Jogging was typically initiated between POW 5 and 12, with the earliest reintroduction at POW 4 and the latest at POW 24, resulting in a mean initiation time of POW 9 [5,14,15,16,17,18,19,20,23,25,26,27,28,29,30,31,32,33,36,37,39,41,43,44,45,46,47,48,50,52,53,57,59,60,62,63,64,65,66,69,70,71,72,73,74,75,76].

Cycling was typically permitted earlier in the rehabilitation process, most commonly between POW 1 and 3, reflecting its role as a low-impact endurance modality. However, some protocols delayed cycling until POW 12, resulting in a mean initiation time of POW 4 [5,14,15,16,17,18,19,21,22,23,24,26,28,29,31,32,35,36,37,40,41,43,45,46,49,51,53,55,56,57,58,59,60,61,62,63,64].

Swimming was less consistently reported and generally introduced between POW 2-6 in those protocols that allowed early aquatic exercise, whereas others delayed or did not specify swimming, sometimes postponing it to POW 24, resulting in a mean initiation of POW 6 [5,14,16,17,18,19,25,26,28,29,30,31,35,36,41,45,52,53,59,62,63,72].

Return to non-contact and contact sport spanned a wide window, with non-contact participation reported from around POW 12 up to 24 [18,33,42,44,68]. Full RTS ranged from approximately POW 3 in isolated early-return protocols to POW 24-48 in time- and criteria-intensive programs, with a mean RTS around POW 15 [5,14,15,16,17,18,19,20,23,25,26,27,28,29,30,31,32,33,36,37,39,41,43,44,45,46,47,48,50,52,53,57,59,60,62,63,64,65,66,69,70,71,72,73,74,75,76,80]. When specified, full RTS depended not only on elapsed time but also on meeting predefined criteria, including full knee ROM, absence of effusion or pain, and restoration of muscle function and functional performance. Common thresholds encompassed quadriceps or quadriceps/hamstring LSI above 70–90%, hop-test LSI above 85–90%, and acceptable hamstring-to-quadriceps ratios (H:Q), illustrating a shift toward criterion-based decision-making rather than strictly time-based clearance.

### 3.6. Key Components of Accelerated Rehabilitation

**Figure 2:**
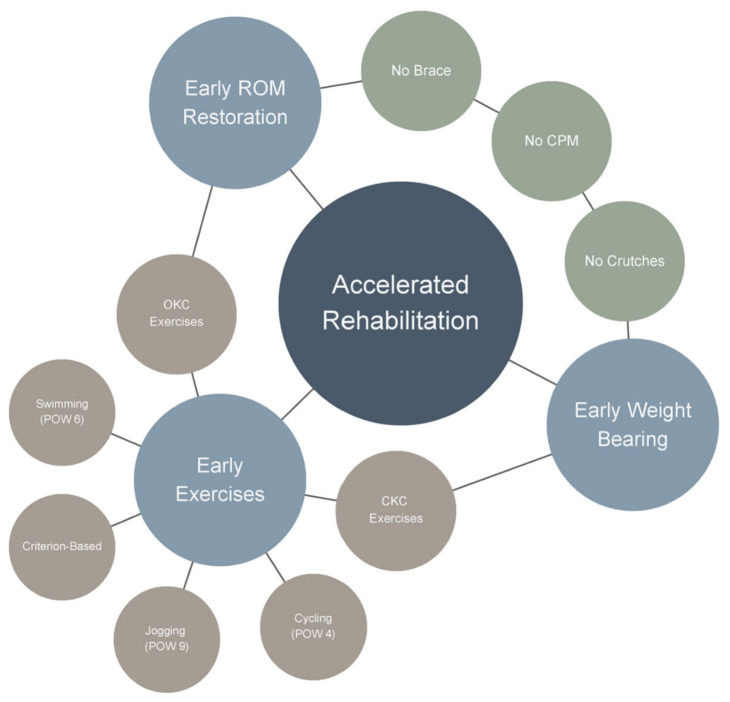
Key Components of Accelerated Rehabilitation. Continuous Passive Motion (CPM), Open Kinetic Chain (OKC), Postoperative Week (POW), and Range of Motion (ROM).

Across the included accelerated rehabilitation protocols, several recurring components emerged, including early restoration of knee ROM, rapid progression to full WB, and early initiation of CKC and OKC exercises while avoiding prolonged immobilization. In many protocols, an accelerated approach was further characterized by the absence of CPM, postoperative bracing, and crutch use, reflecting a shift toward active, function-oriented rehabilitation. These elements were typically implemented within a criterion-based, individualized framework that emphasized symptom-guided load progression, objective strength and functional milestones, and active patient participation rather than rigid time-based restrictions. The schematic in Figure 2 summarizes these key components of the accelerated rehabilitation approach and illustrates how they interact to support early functional restoration and safe RTS after ACLR.

## 4. Discussion

The most important finding of this scoping review is the lack of a universally accepted definition of accelerated rehabilitation following ACLR, despite broad agreement on its core principles and adoption in clinical practice. Across the 68 included studies, accelerated rehabilitation was characterized by early, brace- and crutch-free rehabilitation with rapid restoration of knee ROM, early progression to full WB, early initiation of functional exercises, and avoidance of prolonged immobilization. However, the specific timing, intensity, and frequency of these components varied substantially, highlighting significant heterogeneity in how accelerated rehabilitation is understood and implemented across different healthcare systems and research contexts.

A second key finding is that accelerated rehabilitation protocols consistently prioritize early postoperative goals that directly address common complications following ACLR, particularly arthrofibrosis (including cyclops lesions), quadriceps inhibition, and delayed functional recovery. Nearly all included protocols permitted unrestricted or near-unrestricted knee extension within the first POD, reflecting evidence that early restoration of full extension is critical to prevent flexion contractures, abnormal gait mechanics, and compromised long-term outcomes [5]. This emphasis is strongly supported by contemporary clinical practice guidelines, including the Aspetar Clinical Practice Guideline and earlier evidence-based recommendations by van Melick et al. [81,82].

In contrast, knee flexion was generally progressed more cautiously, most commonly limited to approximately 90° during POW 1-2 before advancing toward full flexion by weeks 4–6. This asymmetric ROM strategy reflects biomechanical evidence demonstrating that knee extension places minimal strain on the ACL graft, whereas deep flexion angles may increase anterior tibial translation and graft loading during the early healing phase [83].

The consistency of this pattern across accelerated protocols suggests a shared underlying biomechanical rationale, even in the absence of standardized terminology. Early progression to WB represents another defining feature of accelerated rehabilitation identified in this scoping review. A substantial proportion of the included studies allowed full WB within the first 48 h postoperatively, and many permitted immediate WB as tolerated. This approach aligns with findings from the MOON working group, which demonstrated that early WB reduces anterior knee pain and facilitates functional recovery without increasing graft failure rates [84]. Similarly, criterion-based rehabilitation models emphasize pain, effusion, and gait quality rather than time-based restrictions as determinants of load progression [85,86]. Because WB and other decisions may be influenced by concomitant injuries, the recently published meniscus rehabilitation consensus can be used to guide more precise recommendations [87,88]. Postoperative knee bracing was another area of marked variability. Although some studies continued to employ short-term bracing, a substantial proportion of accelerated protocols eliminated bracing entirely or restricted its use to the immediate postoperative period. This trend is consistent with systematic reviews demonstrating no clear benefit of routine bracing following isolated ACLR in terms of pain, stability, ROM, or long-term functional outcomes [89]. Increasingly, bracing appears to be reserved for patient comfort, psychological reassurance, or cases involving concomitant injuries rather than as a routine component of accelerated care.

Early quadriceps activation and CKC exercises were nearly universal across protocols, typically initiated within the first POW. CKC exercises are favoured due to their biomechanical profile and functional relevance, promoting quadriceps strength while minimizing anterior shear forces at the knee [90]. Progression to more demanding CKC tasks, including squats, lunges, and step-based activities, generally occurred between POW 2-6, in parallel with improvements in ROM and WB tolerance. This reinforces the concept that accelerated rehabilitation should be understood as optimized rehabilitation rather than simply faster rehabilitation. OKC exercises, particularly knee extension, were introduced with greater variability. Historically delayed due to concerns regarding graft strain, OKC exercises are increasingly incorporated earlier in rehabilitation when appropriately dosed. Criterion-based frameworks proposed by Adams et al. [85] and further refined by Brinlee et al. support the controlled introduction of OKC knee extension within safe ROM, emphasizing its importance for restoring quadriceps strength symmetry [91]. This shift reflects a broader evolution away from blanket exercise restrictions toward evidence-based risk stratification. These findings further emphasize that early restoration of full knee extension should be regarded as a cornerstone of accelerated rehabilitation rather than merely one component of an accelerated protocol. Across the included studies, unrestricted knee extension was one of the most consistently implemented rehabilitation principles, reflecting its critical role in preventing arthrofibrosis, restoring normal gait mechanics, reducing quadriceps inhibition, and facilitating early functional recovery. The remarkable consistency with which early extension recovery was prioritized, despite considerable variation in other rehabilitation components, suggests that this principle represents one of the most robust and evidence-supported characteristics of contemporary accelerated ACL rehabilitation.

The relevance of graft choice to rehabilitation progression warrants particular attention. A systematic review by Zhang et al. focusing on quadriceps tendon ACLR demonstrated that postoperative rehabilitation principles remain largely consistent with other graft types, including early extension restoration and progressive loading, but highlighted graft-specific considerations such as anterior knee symptoms and quadriceps strength recovery [92]. These findings reinforce the need to tailor accelerated rehabilitation protocols to graft-specific biomechanical and biological characteristics rather than adopting a one-size-fits-all approach.

A further important finding of this scoping review is the growing adoption of criterion-based progression models. Many accelerated protocols implicitly or explicitly incorporated objective clinical milestones, such as resolution of effusion, symmetrical ROM, normalized gait, and quadriceps strength to guide progression through rehabilitation phases. This approach represents a fundamental departure from traditional time-based protocols and is now widely regarded as best practice [85,91]. However, inconsistent reporting of these criteria across studies limits direct comparison and complicates evidence synthesis. Psychological readiness and self-management strategies are increasingly recognized as components of successful accelerated rehabilitation [93]. A recent meta-analysis reported that structured psychological interventions improve rehabilitation adherence, confidence, and functional outcomes following ACLR [94]. Complementing this, Foissey et al. showed that regular use of a self-rehabilitation mobile application for more than two weeks significantly reduced extension deficits and the incidence of cyclops syndrome after ACLR [95].

This scoping review highlights substantial variability in rehabilitation delivery, influenced by healthcare infrastructure and different systems worldwide, which have an impact on access to physiotherapy. Survey data from South Africa illustrate how resource constraints may necessitate more conservative or inconsistent rehabilitation approaches despite strong evidence supporting early mobilization [96]. In contrast, publicly funded systems such as the UK’s National Health Service increasingly emphasize early WB and patient-directed exercise progression, aligning well with accelerated rehabilitation principles but placing greater responsibility on patient education and self-efficacy.

Collectively, the findings of this scoping review indicate that accelerated rehabilitation after ACLR is not a single standardized protocol but rather a conceptual approach grounded in early functional restoration, progressive loading, and individualized, criterion-based decision-making. These principles are commonly reported within contemporary rehabilitation literature; however, the absence of a universally accepted definition of accelerated rehabilitation may contribute to variability in study comparisons and influence the development of consistent clinical recommendations.

When interpreting the results of this scoping review, it is important to consider a number of limitations. An important consideration and a key limitation of this scoping review is the historical evolution of the concept of accelerated rehabilitation. The studies included in this review span more than three decades, during which substantial advances occurred in surgical techniques, graft fixation, understanding of graft biology, and postoperative rehabilitation. Consequently, several interventions originally described as components of “accelerated” rehabilitation in the 1990s, including immediate WB, early restoration of full knee extension, and minimization of postoperative immobilization, have progressively become standard elements of contemporary ACL rehabilitation. This historical evolution introduces substantial heterogeneity across the included studies and limits direct comparison of rehabilitation protocols developed in different eras. Accordingly, the term accelerated rehabilitation is inherently relative to the prevailing standard of care at the time a study was conducted, and this temporal heterogeneity should be considered when interpreting the findings of this review. Rather than representing a fixed rehabilitation protocol, accelerated rehabilitation should therefore be viewed as an evolving treatment philosophy aimed at promoting safe and evidence-based functional recovery. Likewise, while criterion-based progression is widely recognized as a hallmark of contemporary rehabilitation, many earlier studies classified as accelerated relied primarily on predefined time-based milestones. Objective progression criteria, such as restoration of symmetrical ROM, minimal joint effusion, normalized gait, quadriceps strength, or functional hop performance, were reported inconsistently and predominantly in more recent publications. Thus, the identification of criterion-based progression as a defining characteristic reflects the evolution of rehabilitation concepts over time rather than a feature consistently implemented across all included studies.

Another important limitation is that this scoping review was intentionally designed to characterize the structural components of accelerated rehabilitation protocols rather than to synthesize clinical outcomes or evaluate treatment effectiveness. Accordingly, studies were included based on their description of rehabilitation protocols rather than the completeness of outcome reporting. As a result, no conclusions can be drawn regarding whether accelerated rehabilitation results in earlier return to sport or competition, improved functional outcomes, or superior long-term clinical results. These findings should therefore be interpreted as describing the organizational principles that distinguish accelerated rehabilitation protocols within the existing literature. The substantial heterogeneity among the included studies further limits direct comparisons between rehabilitation protocols. Differences in concomitant surgical procedures, including meniscal repair, time from injury to surgery, cartilage restoration procedures, and lateral extra-articular augmentation, as well as variation in graft selection, likely influenced postoperative restrictions, weight-bearing progression, ROM limitations, and overall rehabilitation timelines. However, these factors were inconsistently reported and therefore could not be systematically analyzed. In addition, although the inclusion of randomized controlled trials, cohort studies, narrative reviews, surgical technique articles, and other publication types is consistent with the methodology of a scoping review and allowed comprehensive mapping of the available literature, these study designs contribute different levels of evidence. Therefore, the proposed characteristics of accelerated rehabilitation should be interpreted as an evidence-informed synthesis of the current literature rather than a definition derived exclusively from high-level clinical studies.

Second, the main goal of this scoping review was to map and describe the body of existing literature rather than to evaluate study quality or compare results between accelerated and traditional rehabilitation protocols. As a result, there was no formal risk-of-bias assessment, and the findings should not be interpreted as evidence of clinical superiority.

Third, there was significant variation among the included studies in terms of reporting quality, graft types, timing of progression, and rehabilitation content. Quantitative synthesis and direct comparison between protocols were hindered by the inconsistent definition and documentation of “accelerated rehabilitation.”

Only studies with accessible abstracts and full texts were included, and some potentially relevant articles were excluded due to unavailable full texts or language barriers, introducing potential selection bias. In addition, variation in the level of detail reported for rehabilitation protocols may have affected the accuracy of data extraction.

A further limitation of this scoping review is the inability to conduct subgroup-specific analyses based on age or sex. Although younger athletes and female patients are recognized as having higher rates of secondary ACL injuries and distinct neuromuscular recovery patterns, the heterogeneity and inconsistent reporting of demographic data across studies prevented stratified analyses. Additionally, this review did not assess reinjury or graft failure rates in relation to the characteristics of accelerated rehabilitation. Therefore, firm conclusions regarding the safety of accelerated rehabilitation with respect to secondary ACL injury risk, particularly among high-risk groups such as young athletes returning to pivoting sports cannot be drawn.

## 5. Conclusions

Accelerated rehabilitation is commonly characterized by early, brace- and crutch-free rehabilitation, rapid restoration of knee ROM, early full WB, and early initiation of functional exercises. These shared principles reflect a shift toward criterion-based, individualized rehabilitation aimed at optimizing functional recovery while minimizing postoperative complications.

## Figures and Tables

**Table 1:** Patient Characteristics of the Included Studies.

Author	Publication Year	Origin	Study Type	Patients N	Mean Age Year (SD)	Male N	Female N	Patient Characteristics
Shelbourne et al. [5]	1990	USA	Retrospective Comparative Cohort Study	274	NR	147	73	NR
Shelbourne et al. [14]	1993	USA	Retrospective Case Series	33	29.5	26	7	NR
Shelbourne et al. [15]	1995	USA	Retrospective Cohort Study	209	NR	NR	NR	NR
Shelbourne et al. [16]	1995	USA	Retrospective Comparative Cohort Study	143	21.3	100	43	NR
Chapman et al. [17]	1995	Australia	Observational Cross-sectional Study	20	24.5 (3.1)	11	9	NR
MacDonald et al. [18]	1995	USA	Prospective Case Series	40	23.5	34	6	Athletes
Feiring et al. [19]	1996	USA	Cross-sectional Comparative Study	23	NR	NR	NR	NR
Grøntvedt et al. [20]	1996	Norway	Randomized Controlled Trial	100	26	45	55	NR
Shelbourne et al. [21]	1997	USA	Prospective Cohort Study	1057	23.2	765	301	NR
De Carlo et al. [22]	1997	USA	Retrospective Cohort Study	180	27.8	130	50	NR
Bach et al. [23]	1998	USA	Retrospective Cohort Study	103	NR	NR	NR	NR
Mucha et al. [24]	1998	Germany	Prospective Comparative Cohort Study	32	NR	NR	NR	NR
Muneta et al. [25]	1998	Japan	Prospective Comparative Cohort Study	103	24.5 (7.5)	43	60	Athletes
Sauter et al. [26]	1999	Netherlands	Prospective Case Series	50	26	31	19	NR
Howell et al. [27]	1999	USA	Prospective Comparative Study	67	30	47	20	NR
Feller et al. [28]	2001	Australia	Randomized Controlled Trial	65	26.65 (6.2)	47	18	NR
Odat et al. [29]	2001	Jordan	Retrospective Cohort Study	50	NR	50	0	Soldiers
Peterson et al. [30]	2001	USA	Prospective Nonrandomized Study	119	NR	NR	NR	NR
Majima et al. [31]	2002	Japan	Prospective Comparative Study	32	NR	18	14	NR
Beynnon et al. [32]	2005	USA	Randomized Controlled Trial	10	30.4	5	5	NR
Giron et al. [33]	2005	Italy	Prospective Cohort Study	43	29	34	9	NR
Ibrahim et al. [34]	2005	Kuwait	Randomized Controlled Trial	85	22.3	85	0	NR
Pförringer et al. [35]	2005	Germany	Randomized Controlled Trial	46	29	19	27	NR
Salman Ali et al. [36]	2006	UK	Prospective Cohort Study	74	35.1	69	5	NR
Cossey et al. [37]	2006	Australia	Retrospective Case Series	49	27	29	20	NR
Kvist et al. [38]	2006	Sweden	Case-control Study	7	25	5	2	NR
Vadalà et al. [39]	2007	Italy	Randomized Controlled Trial	18	29	13	5	NR
Melikoglu et al. [40]	2008	Turkey	Prospective Cohort Study	98	NR	85	13	NR
Kim et al. [41]	2009	Korea	Retrospective Comparative Study	48	28.65 (9.12)	39	9	NR
Stengel et al. [42]	2009	Germany	Randomized Controlled Trial	54	28.9 (11.3)	35	19	NR
Karasel et al. [43]	2010	Turkey	Prospective Case Series	38	27.6 (6.4)	33	5	NR
Tirmik et al. [44]	2010	Turkey	Prospective Case Series	50	27.4	49	1	NR
Åhlén et al. [45]	2011	Sweden	Retrospective Cohort Study	61	26.5 (7)	34	27	NR
Beynnon et al. [46]	2011	USA	Randomized Controlled Trial	19	29.7	13	6	NR
Janssen et al. [47]	2011	Netherlands	Case-control Study	67	NR	NR	NR	NR
Janssen et al. [48]	2012	Netherlands	Prospective Cohort Study	22	NR	NR	NR	NR
Christensen et al. [49]	2013	USA	Randomized Controlled Trial	15	30.1 (10.5)	8	7	NR
Janssen et al. [50]	2013	Netherlands	Case Series	199	NR	57	43	NR
J. Lee et al. [51]	2013	Korea	Prospective Case Series	10	26.2 (2.68)	5	5	NR
Zhu et al. [52]	2013	China	Observational Retrospective case-controlled Series	30	NR	NR	NR	NR
An et al. [53]	2015	Korea	Prospective Comparative Study	18	27 (6.5)	10	8	NR
Falconer et al. [54]	2015	Australia	Prospective Case Series	111	NR	NR	NR	NR
M. Lee et al. [55]	2016	Korea	Prospective Comparative Study	8	18 (1.71)	NR	NR	Soccer Players
Luo et al. [56]	2016	China	Randomized Controlled Trial	20	39.6 (11.3)	13	7	NR
Gupta et al. [57]	2017	India	Randomized Controlled Trial	20	26.45 (4.7)	20	0	NR
Feyzioğlu et al. [58]	2019	Turkey	Prospective Intervention Study	30	25.83	19	11	NR
Hajouj et al. [59]	2020	Iran	Randomized Controlled Trial	19	24.68 (3.78)	19	0	Athletes
Cristiani et al. [60]	2020	Sweden	Randomized Controlled Trial	80	28.65 (5.9)	61	19	NR
Kurtoğlu et al. [61]	2021	Turkey	Retrospective Cohort Study	72	27.6 (5)	64	8	NR
Hajouj et al. [62]	2021	Iran	Randomized Controlled Trial	15	24.33 (3.68)	15	0	Athletes
Sohu et al. [63]	2022	Pakistan	Randomized Controlled Trial	40	NR	NR	NR	NR
Ebert et al. [64]	2022	Australia	Randomized Controlled Trial	22	24.9 (7.1)	12	10	NR
Jin et al. [65]	2022	China	Randomized Controlled Trial	33	29.06 (6.96)	19	14	NR
Patra et al. [66]	2022	India	Randomized Controlled Trial	40	34	37	3	NR
Török et al. [67]	2022	Hungary	Prospective Cohort Study	54	24 (3)	46	8	NR
Yu et al. [68]	2022	China	Retrospective Observational Study	91	29 (9.15)	54	37	NR
S. Lee et al. [69]	2023	Korea	Retrospective Cohort Study	10	NR	10	0	NR
Deichsel et al. [70]	2023	Germany	Retrospective Cohort Study	33	29.3	25	8	NR
Ozdamar et al. [71]	2024	Turkey	Retrospective Cohort Study	51	NR	50	1	NR
Elabd et al. [72]	2024	Egypt	Randomized Controlled Trial	50	21.92 (1.74)	50	0	Athletes
Mardani-Kivi et al. [73]	2024	Iran	Randomized Controlled Trial	42	NR	34	8	NR
Svensson et al. [74]	2024	Sweden	Randomized Controlled Trial	78	28.5 (5.86)	59	19	NR
Yang et al. [75]	2024	China	Randomized Controlled Trial	45	35.4 (9.6)	21	24	NR
Bairwa et al. [76]	2025	India	Prospective Comparative Study	58	27.8 (5.1)	47	11	NR

Not Reported (NR).

**Table 2:** Summary of Knee Bracing, Range of Motion Recovery Timelines, and Weight-Bearing Status in Accelerated ACLR Protocols.

Author	Knee Brace	Crutches	Knee ROM *Extension*	Knee ROM *Flexion*	Weight-Bearing	
					*Partial*	*Full*
Shelbourne et al. [5,14,16] Chapman et al. [17] Feller et al. [28] Odat et al. [29] Salman Ali et al. [36] Åhlén et al. [45] An et al. [53] Sohu et al. [63]	POW 3-5 (Functional) 1 Year (Competition)	POD 1-2	Unrestricted (POD 2)	90° (POD 2) 110° (POW 2) 130° (POW 5) Full (POW 10)	-	POD 1
Shelbourne et al. [15] Ozdamar et al. [71]	POW 1-2 (0° Flexion)	POD 1-NR	Unrestricted (POD 1)	90° (POW 2) 135° (POW 5)	-	POD 1
MacDonald et al. [18]	POD 0-NR 1 Year (Functional)	Until full ROM achieved	Unrestricted (POD 0)	Unrestricted (POD 0)	-	POD 0
Feiring et al. [19]	NR	NR	NR	NR	NR	POD 2
Grøntvedt et al. [20]	-	NR	Unrestricted (POD 0)	Unrestricted (POD 0)	POD 0	As soon as tolerated
Shelbourne et al. [21] Melikoglu et al. [40]	NR	Optional	Full (POD 2)	90° (POD 0) 110° (POW 2)	-	POD 0
De Carlo et al. [22]	NR	Until normal gait	Unrestricted (POD 0)	90° (POD 0) 110° (POD 7) 135° (POW 4) Full (POW 5)	POD 0	As soon as tolerated
Bach et al. [23]	POW 1-6 (0° Flexion)	Until normal gait	NR	NR	-	POD 0
Mucha et al. [24]	NR	NR	NR	NR	POD 0	POW 1
Muneta et al. [25]	POD 0-3 (30° Flexion)	POW 1-3	NR	NR	-	POD 0
Sauter et al. [26]	-	POW 1-3	Unrestricted (POD 1)	NR	-	POD 1
Howell et al. [27]	-	NR	NR	NR	-	POD 0
Peterson et al. [30]	1 Year	POW 1-3	NR	NR	-	POD 0
Majima et al. [31]	POW 1-6 (Functional)	NR	NR	90° (POD 0)	-	POD 0
S. Lee et al. [69]	POW 1-2 (0° Flexion) Thereafter ambulating	POD 1-NR	Full (POW 1)	90° (POD 5)	-	POD 1
Beynnon et al. [32,46] Gupta et al. [57]	POD 1-NR (20° Flexion) Functional (1 Year)	NR	Unrestricted (POD 8)	Unrestricted (POD 8)	-	POW 2
Giron et al. [33]	NR	NR	Unrestricted (POD 2)	30° (POD 2)	POD 1	POD 3
Ibrahim et al. [34]	POD 0-3	POD 4-NR	Full (POW 8)	60° (POD 4) 90° (POD 14)	POD 4	POD 5
Pförringer et al. [35]	POD 0-3 (0° Flexion)	POD 4-NR	Full (POW 8)	60° (POD 4) 90° (POD 14)	POD 4	POW 8
Cossey et al. [37]	NR	POW 1-2	Unrestricted (POD 0)	Unrestricted (POD 0)	-	POD 1
Kvist et al. [38]	-	POW 1-6	Unrestricted (POD 0)	Unrestricted (POD 0)	-	POD 2
Kim et al. [41]	POW 1-4	NR	Unrestricted (POD 0) Full (POW 4)	Unrestricted (POD 0) Full (POW 1-4)	-	POD 0
Vadalà et al. [39]	NR	NR	NR	90° limited (POW 6)	-	POD 1
Christensen et al. [49] Kurtoğlu et al. [61]	-	NR	Unrestricted (POD 0)	125° (POD 1)	-	POD 2
Stengel et al. [42]	NR	NR	NR	90° limited (POW 6)	-	POD 1
Hajouj et al. [59,62]	NR	POD 4-10	Full (POW 1)	90° (POW 1) 120° (POW 2) 130° (POW 5)	-	POD 1
Karasel et al. [43]	POW 1-2 (0° Flexion) POW 3-4 (Full ROM)	POW 1-2	20° (POW 2) Full (POW 6)	90° (POW 2) 120° (POW 4) Full (POW 6)	POD 1	POW 3
Tirmik et al. [44]	POW 1-3	NR	NR	Full (POW 6)	POD 1	POW 4
Janssen et al. [47,48,50]	-	POW 1-2	Unrestricted (POD 1)	Unrestricted (POD 1)	-	POD 1
J. Lee et al. [51]	NR	NR	Full (POW 2)	Full (POW 4-12)	NR	POW 2
Zhu et al. [52] (Accelerated)	POW 1-4 (0–60° Flexion)	POW 1-12	NR	90° (POW 8)	POD 1	POW 9
Zhu et al. [52] (Aggressive)	POW 1-8	POW 1-4	NR	90° (POW 1) Full (POW 5)	POD 1	POW 3
Falconer et al. [54]	POW 1-3	NR	Unrestricted (POD 0)	Unrestricted (POD 0)	-	POD 0
M. Lee et al. [55]	POW 1-12	POW 1-4	NR	NR	-	POD 2
Luo et al. [56]	POW 1 (0–60° Flexion)	NR	NR	NR	POD 1	POW 6
Feyzioğlu et al. [58]	POW 1-2 (0° Flexion)	POW 1	Unrestricted (POD 0)	90° (POW 1-2) 110° (POW 3) Full (POW 4)	POW 1	POW 2
Cristiani et al. [60]	NR	NR	Unrestricted (POD 0)	Unrestricted (POD 0)	-	POD 0
Ebert et al. [64]	POW 1	POW 1	Unrestricted (POD 1)	90° (POW 1-2) 120° (POW 3)	POD 1	POW 2
Jin et al. [65]	POW 1 (0° Flexion) POW 2-10 (Ambulating)	POD 3-NR	NR	45° (POD 1) 90° (POD 4) 120° (POW 3) Full (POW 6)	POD 3	POW 2
Patra et al. [66]	POW 1-2 (0° Flexion)	NR	Full (POW 6)	45° (POW 1) 120° (POW 4) Full (POW 6)	POD 1	POW 2
Török et al. [67]	POW 1-2	NR	Full (POW 1)	110° (POW 1) 125° (POW 2-6) 135° (POW 6-12)	POD 1	POW 2
Yu et al. [68]	NR	POW 1-4	Full (POW 6)	Full (POW 8)	-	POD 0
Deichsel et al. [70]	-	NR	Unrestricted (POD 5)	Unrestricted (POD 5)	POD 0	POD 5
Elabd et al. [72]	NR	POD 0-6	NR	90° (POD 0) 120° (POW 2)	-	POD 0
Mardani-Kivi et al. [73]	NR -	NR	NR	30° (POD 7) 45° (POW 1) 90° (POW 3) 120° (POW 4) Full (POW 5)	-	POD 1
Svensson et al. [74]	-	NR	Full (POW 4)	90° (POW 4)	-	POD 0
Yang et al. [75]	POW 1 (0° Flexion)	POW 1	Full (POW 1)	90° (POW 1) 120° (POW 2) Full (POW 4)	POD 1	POW 3
Bairwa et al. [76]	POW 1-2 (0–90° Flexion)	NR	NR	Full (POW 4)	POD 1	POW 3

Not Applied (-), Not Reported (NR), Postoperative Day (POD), Postoperative Week (POW), and Range of Motion (ROM).

**Table 3:** Summary of Continuous Passive Motion, Open Kinetic Chain Exercises, Closed Kinetic Chain Exercises, and Additional Interventions in Accelerated ACLR Protocols.

Author	Continuous Passive Motion	Open Kinetic Chain Exercises	Closed Kinetic Chain Exercises	Additional Interventions
Shelbourne et al. [5,14,16] Chapman et al. [17] Feller et al. [28] Odat et al. [29] Salman Ali et al. [36] Åhlén et al. [45] An et al. [53] Sohu et al. [63]	POD 1-NR	Straight leg raises (POD 2) Heel slides (POD 7)	Wall slides (POD 7) Step-ups (POD 7) Calf raise (POD 7) Stairmaster (POW 2) Leg press (POW 2) Quarter squats (POW 2)	NR
Shelbourne et al. [15] Ozdamar et al. [71]	POD 0-NR	Knee extension (POD 0)	Step-ups (POW 3) Calf raise (POW 3) Leg press (POW 3) Lateral shuffles (POW 5)	NR
MacDonald et al. [18]	NR	Straight leg raises (POD 0) Isometric knee extension (POD 0) Knee extension/flexion (POW 8)	Leg press (POW 4) Quarter squats (POW 4) Calf raises (POW 4)	NR
Feiring et al. [19]	NR	Straight leg raises (POD 2) Knee extension (POD 2)	Quarter squats (POD 2) Leg press (POD 2) Calf raises (POD 2) Quarter squats (POW 2) Stairmaster (POW 2) Trampoline (POW 2) Step-ups (POW 2) Lateral shuffles (POW 2)	NR
Grøntvedt et al. [20]	NR	NR	NR	NR
Shelbourne et al. [21] Melikoglu et al. [40]	POW 1-2 (0–0–30°)	Short-arc knee extension (POD 0) Knee flexion (POW 3)	Squats (POW 3) Leg press (POW 3) Step-ups (POW 3) Stairmaster (POW 3)	NR
De Carlo et al. [22]	POD 0-2 (10–0–30°)	Straight leg raises (POD 0) Short-arc knee extension (POD 1) Heel slides (POD 7) Knee flexion (POD 7) Knee extension (POW 2)	Wall slides (POD 7) Calf raises (POD 7) Leg press (POW 2) Step-ups (POW 2) Stairmaster (POW 2) Squats (POW 5)	NR
Bach et al. [23]	POD 0-1	Straight leg raises (POD 0)	Stairmaster (POW 4)	NR
Mucha et al. [24]	NR	NR	Leg press (POW 1) Half squats (POW 1)	NR
Muneta et al. [25]	NR	NR	Half squats (POW 4)	NR
Sauter et al. [26]	NR	NR	NR	NR
Howell et al. [27]	NR	All exercises unrestricted (POW 4)	All exercises unrestricted (POW 4)	NR
Peterson et al. [30]	NR	NR	NR	NR
Majima et al. [31]	NR	Isometric knee extension (POD 0)	Calf raises (POD 7) Step-ups (POW 2) Squats (POW 2)	NR
S. Lee et al. [69]	POD 1 (0–0–50°) POD 2-7 (0–0–90°)	Straight leg raises (POD 1) Isometric knee extension (POD 1) Knee flexion (POD 2)	Quarter squats (POD 1) Half squats (POW 2) Leg press (POW 2) Step-ups (POW 3) Stairmaster (POW 3)	EMS (POD 1-7) Pool walking (POW 3)
Beynnon et al. [32,46] Gupta et al. [57]	NR	Straight leg raises (POW 2) Short-arc knee extension (POW 2) Knee flexion (POW 2) Knee extension (POW 5)	Toe raises (POW 3)	NR
Giron et al. [33]	NR	NR	NR	NR
Ibrahim et al. [34]	NR	NR	NR	NR
Pförringer et al. [35]	NR	NR	NR	NR
Cossey et al. [37]	NR	Knee extension (POW 10)	NR	NR
Kvist et al. [38]	NR	Straight leg raises (POD 0) Knee extension (POW 1)	NR	NR
Kim et al. [41]	NR	Short-arc knee extension (POW 5)	NR	NR
Vadalà et al. [39]	NR	NR	NR	NR
Christensen et al. [49] Kurtoğlu et al. [61]	POW 1 (0–0–30°)	Heel slides (POD 0)	NR	NR
Stengel et al. [42]	NR	NR	NR	NR
Hajouj et al. [59,62]	POW 1	Straight leg raises (POD 1) Knee extension/flexion (POW 2)	Half squats (POW 1) Stairmaster (POW 4)	NR
Karasel et al. [43]	POD 1	Straight leg raises (POD 1) Knee flexion (POD 1)	Wall slides (POD 1) Lunges (POW 4)	NR
Tirmik et al. [44]	NR	NR	NR	NR
Janssen et al. [47,48,50]	POW 1-2 (0–0–90°)	NR	NR	NR
J. Lee et al. [51]	NR	Straight leg raises (POD 3) Knee extension/flexion (POD 3)	Quarter squats (POD 3) Leg press (POW 2) Stairmaster (POW 2) Calf raises (POW 4)	NR
Zhu et al. [52] (Accelerated)	NR	POW 12	POW 8	NR
Zhu et al. [52] (Aggressive)	NR	POW 4	POW 2	NR
Falconer et al. [54]	NR	POW 8	POW 3	NR
M. Lee et al. [55]	POW 1	Straight leg raises (POD 1) Knee extension/flexion (POW 2)	Wall slides (POW 2) Quarter squats (POW 2) Calf raises (POW 2) Leg press (POW 4) Lunges (POW 4) Stairmaster (POW 4) Step-ups (POW 4)	Cryotherapy (POD 1)
Luo et al. [56]	NR	Knee extension/flexion (POW 1) Straight leg raises (POW 2)	Squats (POW 5)	NR
Feyzioğlu et al. [58]	POW 1-4 (0–0–90°)	Straight leg raises (POD 1) Knee extension/flexion (POW 2)	Leg press (POW 2) Quarter squats (POW 2) Half squats (POW 4) Step-ups (POW 4) Calf raises (POW 4)	NMES (POD 1-7)
Cristiani et al. [60]	NR	Straight leg raises (POD 1)	Calf raises (POD 1) Lunges (POW 7) Stairmaster (POW 7)	NR
Ebert et al. [64]	NR	Straight leg raises (POW 2) Knee extension/flexion (POW 3)	Leg press (POW 3) Squats (POW 3) Calf raises (POW 4) Lunges (POW 4) Step-ups (POW 4)	EMS (POD 1)
Jin et al. [65]	NR	NR	NR	NR
Patra et al. [66]	NR	Knee extension/flexion (POW 1) Straight leg raises (POW 2)	Stairmaster (POW 4) Lunges (POW 6)	Aquatic exercise (POW 4)
Török et al. [67]	POW 1-2	NR	NR	EMS (POW 2)
Yu et al. [68]	POW 1 (0–0–50°)	NR	NR	NR
Deichsel et al. [70]	NR	NR	NR	NR
Elabd et al. [72]	NR	Straight leg raises (POD 1) Knee extension (POW 2)	Quarter squats (POW 2) Leg press (POW 2) Stairmaster (POW 4) Squats (POW 8) Lunges (POW 9)	Cryotherapy (POD 1)
Mardani-Kivi et al. [73]	NR	NR	Squats (POW 6)	NR
Svensson et al. [74]	NR	Straight leg raises (POD 1)	Calf raises (POD 1) Step-ups (POW 7) Lunges (POW 7) Stairmaster (POW 7)	NR
Yang et al. [75]	NR	Straight leg raises (POD 1) Knee extension/flexion (POW 3)	Leg press (POW 3) Squats (POW 3) Calf raises (POW 3)	NR
Bairwa et al. [76]	NR	NR	NR	NR

Electrical Muscle Stimulation (EMS), Neuromuscular Electrical Stimulation (NMES), Not Reported (NR), Postoperative Day (POD), Postoperative Week (POW).

**Table 4:** Summary of Return to Jogging, Cycling, Swimming, and Sport in Accelerated ACLR Protocols.

Author	Jogging	Cycling	Swimming	Return to Sport *Non-Contact*	Full Return to Sport *Time/Criteria-Based*
Shelbourne et al. [5,14,16] Chapman et al. [17] Feller et al. [28] Odat et al. [29] Salman Ali et al. [36] Åhlén et al. [45] An et al. [53] Sohu et al. [63]	POW 5	POW 5	POW 5	NR	POW 4 Full ROM No effusion
Shelbourne et al. [15] Ozdamar et al. [71]	POW 5	POW 3	NR	NR	POW 3
MacDonald et al. [18]	POW 10	POW 2	POW 8	POW 16	POW 16 Full ROM Quadriceps strength LSI > 80%
Feiring et al. [19]	POW 12	POW 2	POW 4	NR	POW 16
Grøntvedt et al. [20]	POW 10	NR	NR	NR	Thigh muscles strength LSI > 85%
Shelbourne et al. [21] Melikoglu et al. [40]	NR	POW 2	NR	NR	POW 6
De Carlo et al. [22]	NR	POW 2	NR	Quadriceps LSI > 70%	POW 10
Bach et al. [23]	POW 12	POW 2	NR	NR	POW 24
Mucha et al. [24]	NR	POW 2	NR	NR	NR
Muneta et al. [25]	POW 12	POW 4	POW 4	NR	POW 24
Sauter et al. [26]	POW 6	POW 6	POW 6	NR	POW 12
Howell et al. [27]	POW 8	NR	NR	NR	NR
Peterson et al. [30]	POW 12	NR	NR	NR	POW 24
Majima et al. [31]	POW 5	POW 2	POW 5	NR	POW 24
S. Lee et al. [69]	POW 8	POW 2	NR	NR	POW 16
Beynnon et al. [32,46] Gupta et al. [57]	POW 8	POW 3	POW 3	NR	POW 24
Giron et al. [33]	POW 12	NR	NR	POW 20	POW 24
Ibrahim et al. [34]	NR	NR	NR	NR	NR
Pförringer et al. [35]	NR	POW 12	POW 12	NR	NR
Cossey et al. [37]	POW 6	POW 2	NR	NR	POW 24
Kvist et al. [38]	NR	NR	NR	NR	NR
Kim et al. [41]	POW 12	POW 12	POW 12	NR	POW 24
Vadalà et al. [39]	POW 12	NR	NR	NR	POW 16
Christensen et al. [49] Kurtoğlu et al. [61]		POW 12	NR	NR	POW 8-24
Stengel et al. [42]	NR	NR	NR	POW 12	POW 24
Hajouj et al. [59,62]	POW 8	POW 3	POW 3	NR	Full ROM No Pain and swelling Quadriceps/Hamstring strength and Hop test LSI > 85% H:Q ratio < 15%
Karasel et al. [43]	POW 12	POW 2	NR	NR	Quadriceps/Hamstring LSI > 85%
Tirmik et al. [44]	POW 12	NR	NR	POW 24	POW 36
Janssen et al. [47,48,50]	POW 8	NR	NR	NR	POW 16
J. Lee et al. [51]	NR	POW 2	NR	NR	POW 36
Zhu et al. [52] (Accelerated)	POW 24	NR	POW 24	NR	POW 48
Zhu et al. [52] (Aggressive)	POW 8	NR	POW 8	NR	POW 12
Falconer et al. [54]	POW 8	NR	NR	NR	POW 24
M. Lee et al. [55]	NR	POW 2	NR	NR	NR
Luo et al. [56]	NR	POW 1	NR	NR	NR
Feyzioğlu et al. [58]	NR	POW 4	NR	NR	NR
Cristiani et al. [60]	POW 9	POW 2	NR	NR	NR
Ebert et al. [64]	POW 10	POW 3	NR	NR	POW 36 Full ROM Quadriceps/Hamstring strength and Hop test LSI > 90%
Jin et al. [65]	POW 10	NR	NR	NR	NR
Patra et al. [66]	POW 14	NR	NR	NR	POW 24
Török et al. [67]	NR	NR	NR	NR	POW 16
Yu et al. [68]	NR	NR	NR	POW 24	NR
Deichsel et al. [70]	POW 6	NR	NR	NR	POW 32
Elabd et al. [72]	POW 8	POW 3	POW 3	NR	POW 16
Mardani-Kivi et al. [73]	POW 12	NR	NR	NR	POW 24
Svensson et al. [74]	POW 9	POW 2	NR	NR	NR
Yang et al. [75]	POW 12	POW 2	NR	NR	POW 16
Bairwa et al. [76]	POW 12	NR	NR	NR	POW 36 Hop test LSI > 90%

Hamstring-to-Quadriceps Ratio (H:Q), Limb Symmetry Index (LSI), Not Reported (NR), Postoperative Week (POW), and Range of Motion (ROM).

## Data Availability

All relevant data extracted and analyzed during this review are fully presented within the article, including in the corresponding tables and figures.

## Supplementary Materials

The following supporting information can be downloaded at: https://www.mdpi.com/article/10.3390/medicina62071348/s1, Preferred Re-porting Items for Systematic reviews and Meta-Analyses extension for Scoping Reviews (PRISMA-ScR) Checklist [12].

## Abbreviations

The following abbreviations are used in this manuscript:

ACL	Anterior cruciate ligament
ACLR	Anterior Cruciate Ligament Reconstruction
CKC	Closed Kinetic Chain
CPM	Continuous Passive Motion
EMS	Electrical Muscle Stimulation
H:Q	Hamstring-to-Quadriceps Ratio
JBI	Joanna Briggs Institute
LSI	Limb Symmetry Index
N/A	Not Applicable
NMES	Neuromuscular Electrical Stimulation
NR	Not Reported
OKC	Open Kinetic Chain
POD	Postoperative Day
POW	Postoperative Week
PEMF	Pulsed Electromagnetic Field
PRISMA-ScR	Preferred Reporting Items for Systematic reviews and Meta-Analyses Extension for Scoping Reviews
ROM	Range of Motion
RTS	Return to Sport
WB	Weight-Bearing
